# Empirical Study on Hospitalist System: A Value Creation Perspective

**DOI:** 10.3390/healthcare12100953

**Published:** 2024-05-07

**Authors:** Liang-Hsi Kung, Yu-Hua Yan

**Affiliations:** 1 Department of Nursing, College of Medicine, National Cheng Kung University, No. 1, University Road, Tainan City 701, Taiwan; 2u0635@mail.tmh.org.tw; 2 Tainan Municipal Hospital (Managed by Show Chwan Medical Care Corporation), No. 670, Chongde Rd., East District, Tainan City 701, Taiwan

**Keywords:** hospitalist system, value creation, participation motivation, participation behavior

## Abstract

This study investigates the impact of hospitalist system awareness, motivation, and behavior on value creation within the healthcare context of Taiwan. As population aging and the prevalence of chronic diseases continue to rise, accompanied by increased medical resource consumption, the Taiwan Ministry of Health and Welfare introduced the hospitalist system. Despite its implementation, the number of participating hospitals remains low. Using a questionnaire survey conducted from October 2021 to March 2022, data were collected from medical teams involved in the hospitalist system. A total of 324 valid questionnaires were analyzed. The results reveal that hospitalist awareness positively influences participation motivation (β = 0.846, *p* < 0.001), which subsequently impacts participation behavior positively (β = 0.888, *p* < 0.001). Moreover, participation behavior significantly contributes to value creation (β = 0.869, *p* < 0.001), along with the direct effect of awareness (β = 0.782, *p* < 0.001) on value creation. In conclusion, the successful promotion and implementation of the hospitalist system rely heavily on the support and active participation of medical staff. Effective interactions and comprehensive information dissemination are essential for maximizing healthcare value creation.

## 1. Introduction

The global demographic landscape is undergoing significant transformation with the increase in the elderly population, a phenomenon referred to as population aging. Projections indicate a rise from 12% in 2015 to 22% by 2050 [1], accompanied by a heightened prevalence of chronic diseases, increased consumption of medical resources, and congested emergency departments [2,3]. In response, the Taiwan Ministry of Health and Welfare introduced the hospitalist system, drawing inspiration from its successful implementation in the United States [4].

Initiated in 2015 as the “Specialized General Medicine Attending Physician Care System Promotion Plan”, later renamed the “Hospitalist System Promotion Plan” in 2017, and further refined into the “Hospitalist and Transitional Care Program” in 2020, these initiatives aimed to optimize physician manpower utilization amidst reduced resident physician working hours mandated by the “Labor Standards Act” of 1 September 2019 [4].

While the hospitalist system shares common objectives internationally, such as enhancing medical management efficiency and quality, its motivations and implementations vary across countries. In the United States, the system aims to expedite hospital patient turnover, reduce costs, and improve quality [5,6,7], while South Korea focused on alleviating waiting times and enhancing patient satisfaction [8,9,10]. Similarly, the United Kingdom and Canada faced distinct challenges, including managing complex patient conditions and physician shortages [5,6,7].

Despite its widespread adoption, empirical studies on the hospitalist system’s impact on value co-creation remain limited. This study seeks to address this gap by exploring the value co-creation model within the hospitalist system’s context. Building upon prior research, we aim to investigate how awareness, motivation, and behavior regarding the hospitalist system influence value creation. Specifically, we will examine the correlation between medical team members’ cognition of the hospitalist system and their participation motivation, and the subsequent impact on participation behaviors and value creation.

In summary, this study aims to contribute to the growing literature on the hospitalist system by elucidating its implications for value co-creation. By empirically examining cognitive, motivational, and behavioral dynamics within healthcare teams, we aim to deepen understanding of how the hospitalist system fosters collaborative value creation in patient care delivery.

## 2. Theoretical Background

### 2.1. Hospitalist System

Taiwan’s aging population and the increasing prevalence of chronic diseases pose significant challenges to the healthcare system. In response to this trend, the Ministry of Health and Welfare implemented the hospitalist system in 2015 [4]. This system aims to improve medical management efficiency and quality by incentivizing hospitals to establish dedicated wards staffed by attending physicians who specialize in holistic healthcare. Various hospitals in Taiwan have adopted different models of the hospitalist system based on their specific needs and objectives.

For instance, Linkou Chang Gung Hospital introduced hospitalists in integrated internal medicine wards in 2003 to address a shortage of resident physicians [11]. Similarly, National Taiwan University Hospital established trauma medicine hospitalist wards to alleviate congestion in the emergency department and provide comprehensive care for patients with complex conditions. Additionally, Taichung Veterans General Hospital established an internal medicine hospital ward to enhance multidisciplinary shared care and overcome limitations associated with organ-based diagnosis and treatment [11].

Successful implementation of the hospitalist system relies on trained professional staff, effective management, and comprehensive integration of services [12,13,14]. Factors contributing to integration success include the presence of interdisciplinary team models, sufficient time to reduce cultural differences, and minimal business competition in the region. Conversely, challenges such as the lack of a bidirectional referral system, inadequate coordination of care, and differences in care objectives can hinder integration efforts [15,16].

In Taiwan, there are two main types of hospitalist care models: those where the hospitalist attending physician assumes full responsibility for patient care, and those where care is provided by a mix of dedicated and specialist attending physicians [4]. Despite the benefits of the hospitalist system, challenges such as resistance to change and the need for organizational support persist.

In summary, the hospitalist system in Taiwan represents a significant step toward improving healthcare quality and efficiency. However, ongoing efforts are needed to address challenges and ensure successful integration across healthcare institutions.

### 2.2. Holistic Healthcare

Traditionally, physicians and medical teams have always led hospital medical services, which is a major obstacle to patient-centric medical care [17]. Holistic healthcare is a medical care model. The former Department of Health (currently Ministry of Health and Welfare) promoted “holistic health care plan” in 2005 and proposed the term of “holistic health care” that not only emphasized providing correct and effective prevention methods before a disease occurs and providing patient-centric medical care but also emphasized correct and dignified rehabilitation and support after patients fell sick [18]. 

Holistic healthcare follows the principles of “holistic”, “meticulous”, and “fair” so that patient care can achieve both depth and breadth. 17 “Holistic” refers to an integrated relationship among physiology, psychology, and society. When one becomes sick, his/her psychological and social functions and emotional adaptation may change. The patient is considered as a “whole” to observe the problems encountered and not only focus on the disease. More specifically, when a patient encounters a disease problem, he/she is used as a starting point for in-depth understanding of his/her physiological, psychological, and social aspects, which requires the medical team to work together to provide appropriate services [19,20]. “Holistic health care” also focuses on establishing physician–patient relationships and good communication skills and emphasizes understanding the patient’s and family’s treatment expectations and adjustment difficulties of the disease.

Bullington and Fagerberg [21] opined that holistic healthcare is a fuzzy concept and is usually an approach that satisfies the patient’s physical, mental, and emotional needs and intervention measures [22]. Owing to improvements in medical levels and increase in the complexity of patients’ conditions, medical staff will encounter more and more patients with multiple diseases or problems (physical, mental, spiritual, and social). At the same time, the satisfaction of these healthcare recipients and providers toward the entire medical service is decreased. Therefore, the need for interdisciplinary team cooperation to provide holistic healthcare has gradually increased [23]. The concept of holistic healthcare is to view the patient as a whole when caring for them and not separate them into parts. In addition, the needs of the patients, including physiological, psychological, spiritual, and social aspects, are viewed as a whole, and the patient’s needs and values are used as a direction for clinical decisions [24]. Providing holistic healthcare can help eliminate the fear of people toward diseases and treatments [25].

Since Taiwan implemented holistic healthcare, previous studies also found that physicians and medical staff generally believe that the various items of holistic healthcare are important, and that awareness is high. However, there is still room for improvement in actual implementation. This may be because there is insufficient willingness to carry out holistic healthcare, insufficient team manpower, or differences in service department ownership [26,27]. “Awareness” refers to new knowledge acquisition and usage of past knowledge, including perception, memory, and thinking. Chang [28] pointed out that awareness refers to the psychological process of recognition and understanding of things via conscious activities that include complex psychological activities, such as perception, imagination, identification, inference, and judgment. Festinger [29] stated that awareness is everything perceived by an individual. Cognitive dissonance occurs when an individual shows conflicting attitudes and behavior toward an event, resulting in discomfort [30]. Alden, Friend, and Chun [31] also showed that the possible barriers are lack of awareness, lack of resources, and limited physician time to learn. When physicians lack understanding and vigilance toward disease awareness differences, it decreases the patient-centric level of medical services and increases the risk of medical dispute [32]. Therefore, prompt discovery and salvaging awareness differences in the integrated healthcare system is an important issue.

### 2.3. Awareness, Participation Motivation, and Participation Behavior

“Health Professions Education: A Bridge to Quality” published by the US Institute of Medicine in 2003 emphasized that medical staff must possess five core competencies, of which there was particular emphasis that “interdisciplinary team care” is critical to integrated holistic healthcare [33] and is considered an essential criterion in today’s healthcare [34]. Interdisciplinary team cooperation can provide more complete holistic healthcare for patients and simultaneously allow professional staff to continuously grow during work and improve healthcare quality [35]. The final objective is to encourage patients to quickly recover and return to normalcy [36,37,38].

Many healthcare organizations require medical institutions to propose and implement specific methods to improve collaborating team training methods to enhance patient safety [39]. In order to avoid errors or negligence, the medical sector has been promoting “interdisciplinary team collaborative care education” in recent years, encouraging good communication between specialties and decreasing barriers to opinion exchange to more efficiently solve the problems in team collaboration and promote smooth operation in the patient care pathway [40]. Class culture is an important factor of poor team collaboration and poor communication in healthcare systems [41], and human errors and insufficient team training are often the main causes of accidents [42]. Therefore, interacting with a respectful attitude, mastering team collaboration knowledge and skills, knowing how to correctly approach the subject and provide timely help when needed, constructing an information system for the reorganization of healthcare procedures, and continuous strengthening of care guidance awareness and training for medical staff can allow win–win maximum benefits for physicians and patients, thereby improving holistic healthcare quality [43]. 

Davis et al. [44] conducted a literature review and summarized the factors that affect participation behavior in healthcare quality and patient safety. They found five factors, namely patient-related (e.g., demographic characteristics), illness-related (e.g., illness severity), healthcare professional-related (e.g., healthcare professionals’ knowledge and beliefs), healthcare setting-related (e.g., primary or secondary care), and patient safety (e.g., hand washing). Legare and Thompson-Leduc [45] performed a systematic review study and found that most participants in clinical care practice were physicians (89%) and that the other medical staff accounted for a minority. Ennew and Binks [46] classified participation behavior as (1) information sharing: sharing information with services ensures that the services provided can satisfy the customer’s needs; (2) responsible behavior: the customer plays the role of some employees, which is mostly presented in behaviors such as taking responsibility for supervision and compliance; and (3) personal interaction: interpersonal relationships in interactions such as mutual trust and cooperation.

Most medical-related adverse events are correlated with the lack of communication and cooperation within the team [47,48]. In actuality, medical team collaborative care needs to be learned and learning must include knowledge, skills, and attitudes. 64 With regard to collaborative relationships among interdisciplinary medical team members in healthcare systems, there are also differences in the attitudes of team members and social workers: physicians play the main role of a healthcare provider [49] and medical team members play a synergistic role in medical cooperation [50]. However, the physician may sometimes avoid cooperating with medical team members, which also shows [51] that physicians tend to rely on themselves and not cooperate with other professional staff. This demonstrates that there are differences in the motivational intensity for team cooperation in the interdisciplinary team. Furthermore, it affects the ability of medical team members in playing their roles and functions, and personality characteristics will affect the team cooperation capacity of every member [41]. Therefore, resource integration will not occur without the participation of actors and value co-creation cannot be achieved, demonstrating the importance of participation motivation.

### 2.4. Value Co-Creation

The basic logic of value creation strategy mainly originates from the concept of value-based strategy proposed by Brandenburger and Stuart [52]. This strategy means that as organizations have unique competitive advantages in specific value activities or possess unique resources or capabilities, they should focus on their own specializations and division of labor and separately create their greatest added value. Traditional value creation is based on the premise that companies are value creators [53]. In this model, value is created by the company alone, and the company alone decides what kind of value is to be provided to the consumers. Subsequently, Porter proposed value chain in 1985 [54], which emphasized that company value activities are classified as support activities and primary activities and that products and services form the basis of value. Therefore, related studies focused on technical, product, and manufacturing process innovation [55]. However, the study of Stähler [56] indicated that value mainly originates from two major groups, namely customers and value partners. The researcher believed that value does not originate from existing products but is created when the needs of customers and external partners are met. With improvements in the knowledge of consumers and stakeholders, value creation members will create value in the process of providing product services and two or more actors will carry out value co-provision activities [53,57]. Hence, it can be seen that modern value management is not traditionally created by manufacturers alone and that all members, customers, and partners in the value network may become value co-creation members.

Strategy researchers [53] and marketing researchers [58] separately conducted a series of value co-creation (VCC)-related studies in 2000 and proposed that “all value is co-created” and that value can only be used and experienced by the consumers (users) to demonstrate its benefits. In the last decade, many researchers have focused on value co-creation-related topics and examined the (1) significance, content, and concept of value co-creation [59,60,61,62]; (2) formation, driving, and management of value co-creation [63,64,65,66,67]; and (3) application studies in service systems, healthcare, and B2B [64,68,69,70]. On the contrary, strategy researchers have published three books, namely “The Future of Competition: Co-Creating Unique Value with Customers” [53], “The Power of Co-Creation!” [71], and “The Co-creation Paradigm” [72]. The authors proposed the driving factors (elements) and framework of value co-creation by summarizing many practical cases and organizing the concept and practice of value co-creation.

Value co-creation actions involve the collection and coordination of important stakeholders, and there is a high interdependent relationship between the efforts and actions of individual stakeholders. With regard to the meta-organization of stakeholders in this study, every member has a collective goal of sharing. However, different members are independent, and their motivation and interests for participating in the collective action of value co-creation are not the same [73,74]. Therefore, the lack of cooperation between stakeholders, lack of effort, and enjoying the fruits of others’ labors are generally very common phenomena. This even causes individual stakeholders to lack incentives to continue remaining in this co-creation system [75,76], thereby resulting in the disintegration of collective co-creation actions. Therefore, value is generated by the use of products or services by customers and is subjectively determined by individuals; value varies from person to person and from scenario to scenario and is related to the experiences of the co-creation participants and mental model [77].

“All value is co-created” and “The organization can be considered as a system consisting of major stakeholders” are used as premises so as to let these major stakeholders be willing to stay in their constructed system, actively work hard, provide commitments [76], and attract major stakeholders [72], who are also known as co-creators, to enter and stay in a co-creation system or organization, invest complementary assets specific to the system, and provide fair and reasonable value distribution to the co-creators. In addition, promotion and implementation are dependent on support and participation by hospital professional staff to exert their greatest effects. Good interactions and sufficient information dissemination are required to create the greatest healthcare value.

## 3. Method

### 3.1. Research Framework

This study combines hospitalist system awareness, participation motivation, and participation behavior to examine their effects on value creation in healthcare teams. This study developed a theoretical framework specific to it based on the aforementioned introduction and study objectives. The measurement variables in the study framework include one exogenous variable (hospitalist awareness) and three endogenous variables (hospitalist participation motivation, hospitalist participation behavior, and value creation).

### 3.2. Research Model and Hypotheses 

Various study hypotheses were proposed based on the objectives of this study and study framework content, and their contents are described as follows:

**Hypothesis** **1.** *The higher the hospitalist awareness of the actor, the greater its participation motivation for interdisciplinary team cooperation.*

**Hypothesis** **2.** *The higher the participation motivation of the actor for the hospitalist system, the greater its participation behavior.*

**Hypothesis** **3.** *The higher the participation motivation of the actor, the greater the value created.*

**Hypothesis** **4.** *The higher the hospitalist awareness of the actor, the greater its value creation.*

### 3.3. Research Tools

This study mainly employed questionnaires for data collection. Therefore, during the design process, rigorous questionnaires from Taiwanese and overseas researchers were first collected and used as a basis for the development of questionnaires and questions for this study. The questionnaire used in this study has five parts: (1) general information; (2) hospitalist system awareness; (3) hospitalist system participation motivation; (4) hospitalist system participation behavior; and (5) value creation. For questionnaire responses, all questions were measurement items except for demographic variables, and the 5-point Likert scale was used. The higher the score, the greater the degree of awareness, where five points means extremely agree and one point means extremely disagree (See Appendix A). 

As regards the expert content validity testing, this study invited five experts (one hospital administrator, one director of a clinical medical department, one director of a nursing department, and two physicians with professor qualifications at the level of vice president or higher) to evaluate the original form. The appropriateness of the written descriptions of each item in the scale was graded. During the process, the experts put forward suggestions anonymously, and the researchers collected and measured the unanimous opinions of the expert group and conducted repeated discussions with the expert group. The score was based on a 5-point scale, with 1 indicating “strongly disagree”, which means that the question should be deleted; 2, “disagree”, indicating that the question is not applicable; 3, “normal”, indicating that the question can be retained, but a relatively large revision is required; 4, “agree”, indicating that the topic is retained, but a slight modification is still required; 5, “strongly agree”, indicating that the topic is retained, and no modification is required. At each time, the researchers revised the content of the scale according to the opinions put forward by the experts until all the experts reached a consensus on the review results.

The content validity index (CVI) of the questionnaire used in this study was measured to establish its content validity. As the measurement standard, the CVI of each question must be >0.8, which is used as the standard of the reserved question. The review of the content validity by experts shows that the CVI ranges from 0.95 to 1.00, and the average CVI is 0.975.

In addition, Harman’s single-factor test was used to assess possible common method variance (CMV). The analysis results showed that the first factor could explain 28.76% of the total variance. As no single-factor solution occurred and the first factor did not consider most of the variation, it means that the total variance explained was not >50%. Therefore, CMV was not found in this study measurement. The questionnaire contents are described as follows:

(1) General information: includes type of inpatient care, age, sex, marital status, education level, position, and hospital appraisal grade.
(2) Variable scales: Hospitalist system awareness mainly measures whether the hospital medical team member understands the concept of holistic healthcare and has awareness of holistic healthcare (including whether he/she previously received holistic healthcare training), whether he/she understands holistic healthcare communication techniques, whether he/she understands the treatment expectations and adjustment difficulties of the patient and family (physiological, psychological, spiritual, and social aspects are considered as a whole), whether he/she can respect and respond to the patient’s needs in guiding all clinical decisions, and whether he/she possesses holistic healthcare competency. This scale was designed by referring to previous researchers [10,17,18,26,27], and all 13 questions were measurement questions.

Participation motivation mainly measures the degree of participation motivation in interteam cooperation and includes whether participation in interteam cooperation can promote interpersonal relationships, whether participation in interteam collaboration can obtain mutual recognition and respect, whether participation in interteam collaboration can meet treatment expectations, whether participation in interteam collaboration can share treatment experience, and whether participation in interteam collaboration can provide disease-related knowledge (patients). The theses of researchers [78,79] were mainly used as a reference for the design of this scale, which contains eight measurement questions.

Participation behavior mainly measures the degree of participation behavior for interteam cooperation, including participation in medical behavior, communication and trust, problems solved by teamwork, information sharing, responsible behavior, and interpersonal interactions. The theses of researchers [40,41,42,46] were mainly used as a reference for the design of this scale, which contains eight measurement questions.

Value creation, which mainly measures whether the hospitalist system can create medical value for the cooperation of medical teams. The design of the scale mainly refers to the theoretical discussion of scholars [43,61,62,80,81], and extends the measurement items of the hospitalist system with a total of 14 items. Topics include the following: the hospitalist system can jointly provide patients with more complete medical care, improve the common values of care, reduce medical disputes, enhance the harmonious relationship between doctors and patients, improve service satisfaction, take medical quality and patient safety as the core value of medical care, enhance team cooperation, create lean medical care, create effects for stakeholders (government, hospitals, and patients), etc.

### 3.4. Demographics of Respondents 

For formal data collection, this study selected hospitals that are subsidized by the Ministry of Health and Welfare of Taiwan to implement the hospitalist system as the research scope. The Ministry of Health and Welfare announced in 2021 that the subsidy application qualifications must be for “physicians” for hospital evaluation and teaching hospital evaluation, who were qualified for the occupation category”, and at least two medical departments of the main responsible hospital can apply. After review, 15 funding subsidies were finally approved in 2021. Among them, 10 hospitals were rated as medical centers and 5 as regional hospitals. Accordingly, this study recruited members of the medical team participating in the hospitalist system in these 15 hospitals.

In this study, the individual was taken as the unit of analysis, and a structured questionnaire was filled in anonymously. Before the survey, the interviewers confirmed the survey time with the window of the evaluated hospital. The interviewers were trained, familiarized with the interview procedures, questionnaire contents, and related precautions, and practiced the question and answer methods repeatedly to avoid deviations when the interviewers provided assistance. Before enrollment, the purpose, methods, and procedures of the study were explained to the research participants, and the participants were finally enrolled only after providing verbal consent. To protect the privacy of the research participants and their personal data, the evaluation was conducted anonymously. The 450 questionnaires (30 per hospital) were given out in this study. From October 2021 to March 2022, questionnaire contacts and distribution were carried out. After removing the invalid questionnaires with answering patterns and incomplete answers, 324 valid questionnaires were collected, and the valid questionnaire response rate was 72%.

### 3.5. Ethics Review

This study was reviewed and approved by the Institutional Review Board of Show Chwann Memorial Hospital, and the review number was 1091101.

### 3.6. Data Analysis Methods

After the collected valid questionnaires were coded, SPSS 21.0 statistical software was used for descriptive statistics and reliability analysis, AMOS 21.0 was used for structural equation modeling (SEM), and SEM was used to analyze and validate the various hypotheses proposed in this study.

## 4. Analysis and Results

### 4.1. Demographics of Respondents 

The statistical analysis results of the general information of 324 medical staff in this study showed that the mean age was 38.1 years (standard deviation: 9.3 years). The majority of subjects were women (*n* = 310, 96.7%), single (*n* = 194, 59.9%), and had an education level of university degree (*n* = 273, 84.3%). Additionally, a significant portion of the participants engaged in the mixed care model for inpatient care (*n* = 251, 77.5%), held a hospital accreditation level of medical center (*n* = 231, 71.3%), and practiced as attending physicians (*n* = 42, 13%) (see Table 1).

### 4.2. Measurement of Confirmatory Factor Analysis

Measurement model analysis and structural model analysis were included in the SEM. Firstly, at the measurement model analysis stage, (1) individual item reliability, (2) composite reliability (CR), and (3) average variance extracted (AVE) were used based on the recommendations of Bagozzi and Yi [82]. With regard to the reliability of individual items, the factor loadings of all variable measurements in this study model were between 0.518 and 0.834, which were all >0.5 and statistically significant (*p* < 0.05). In the reliability analysis, the Cronbach’s alpha values of all variables in this study were between 0.973 and 0.982, which met the standard of Hair et al. [83] that the Cronbach’s alpha of the scale must be >0.7 to be acceptable. The CR of the latent variables was between 0.790 and 0.95, which met the standard recommended by Fornell and Larcker [84] that the CR must be >0.6. The AVE of the latent variables was between 0.321 and 0.588, which met the standard recommended by Fornell and Larcker [84] that the AVE must be >0.3. In summary, the above results showed that the internal consistency of this study questionnaire was good, it had high reliability, and that the questionnaire structure was acceptable. Table 2 shows the measurements and confirmatory factor analysis (CFA).

A CFA was used to evaluate the measurement models for the validity and reliability constructs. This study used five measures to assess the goodness of fit of the CFA: all the measures met their threshold values, implying that the model was fit for estimation. Table 3 shows the overall fit of the research model.

### 4.3. Structural Model Analysis

**Figure 1:**
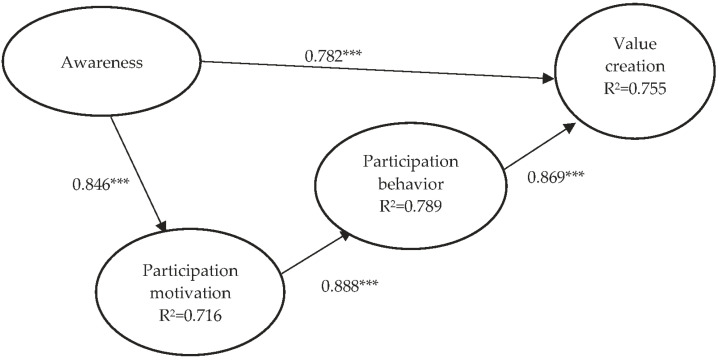
Causal path diagram. (*** denotes *p* < 0.001).

Structural model analysis was mainly used to validate the study framework and describe the explanatory power of the overall model. In this study, AMOS 21.0 was used for analysis, and standardized coefficients were used as the path coefficients to validate the four hypotheses of the study model. The analysis results showed that the structural model path analysis coefficients (as shown in Table 4) were as follows: hospitalist awareness had positive effects on participation motivation (β = 0.846, *p* < 0.001); participation motivation had positive effects on participation behavior (β = 0.888, *p* < 0.001); participation behavior had significant positive effects on value creation (β = 0.869, *p* < 0.001); awareness also had significant effects on value creation (β = 0.782, *p* < 0.001); and hypotheses 1–4 were all supported. Table 4 shows the study hypotheses supported by empirical data. Figure 1 shows the explanatory power of the various endogenous latent variables in the entire model. Standardized coefficients were used in the path values of various dimensions. In the study model, the variance explanatory powers of various endogenous latent variables on the entire model (R^2^) were as follows: the R^2^ of hospitalist participation motivation was 71.6% (0.716); the R^2^ of hospitalist participation behavior was 78.9% (0.789); and the R^2^ of value creation was 75.5% (0.755). Figure 1 shows the study model paths.

## 5. Discussion and Implication

The core spirit of the hospitalist system is patient-centric integrated medical care, improving medical quality, strengthening disease management for patients with high medical resource use and multiple comorbidities to decrease their frequency of outpatient, emergency department, and inpatient visits, and improving the efficient use of medical resources. The primary objective of this study is to examine the effects of hospitalist system awareness, participation motivation, and participation behavior on value creation and to further evaluate the effects of hospitalist system awareness and value creation. The empirical study results showed that the holistic medical value creation model has good explanatory power and that it can be used as a reference for national policy formulation and hospital management.

### 5.1. Management and Practical Implications

This study investigated the relationship of hospitalist system awareness, participation motivation, and participation behavior with value creation. The study results showed that hospitalist system awareness has positive effects on participation motivation, and the hypothesis proposed in this study was supported. Past researchers also pointed out that hospitalists not only treat patient’s diseases but also use empathy to understand the patient’s mental adjustment and living conditions and feelings of family members [85]. Furthermore, the results of this study showed that participation motivation was increased when hospitalist system awareness was increased. Previous studies too found that physicians and medical staff generally believed that the various items of holistic healthcare are important and that awareness is high. However, there is still room for improvement in actual implementation [26,27].

Moreover, the study results indicated that hospitalist system participation motivation has significant positive effects on participation behavior. The medical team must work together to reach the level of the hospitalist. Therefore, physicians, nurses, pharmacists, social workers, rehabilitation therapists, psychologists, and other professionals play crucial roles and have the same value in the team. Previous studies also alluded that the hospitalist system forms the basis for teamwork. Participation behavior also shares information with the service providers to ensure that the provided services satisfy the customer’s needs, and that mutual trust and cooperation are built via interactions [36,46]. Therefore, strong hospitalist system participation motivation has significant positive effects on participation behavior. 

In addition, hospitalist promotion and implementation are dependent on the support and participation of hospital professional staff to exert its greatest effects. Good interactions and sufficient information dissemination are required to create the greatest healthcare value. The empirical study results demonstrated that hospitalist system awareness and participation behavior have significant positive effects on value creation, which is consistent with the viewpoint of researchers [75,81]. The findings signified that the value creation results are enhanced when holistic health team members exhibit high hospitalist system awareness and participation behavior. Previous researchers also stated that value co-creation is achieved via the collective coordination of organization members under a fixed common goal, resource sharing and integration, and shared/collective values. At the same time, it also increases the value and connections of the members, thereby promoting value co-creation by the participants [75,81,86,87]. Therefore, providing complete holistic healthcare services will aid in value creation in an aged society.

### 5.2. Theoretical Implications

Interactions are vital between mutually dependent stakeholders [88,89]. However, how should interactions be started? This study found that formulating a system or rules can create interactions and that the core of value creation is interactions and continuous interactions. This is similar to what Laud and Oswald [90] and Sorensen et al. [91] emphasized, and value creation has attracted the attention of the academia. Co-creation is an outstanding viewpoint in the marketing field and is acknowledged as a contemporary revolution in value creation [92,93]. Interaction experiences and processes are used to achieve a win–win situation for the hospital and the patients. This study has expanded the systematic research on value creation and has extended value creation research to the hospitalist system so that medical teams can better understand the patient’s needs.

## 6. Limitation

This study’s gender distribution, with significantly fewer male participants (14) compared to females (310), raises concerns about representativeness. Factors contributing to this disproportion could include demographic characteristics or challenges in male participant recruitment. Addressing this limitation, we recommend exploring targeted recruitment strategies or incentives to enhance male participation. Additionally, reliance on self-reported questionnaires may introduce response bias, affecting data accuracy and reliability. Future studies could mitigate this by incorporating objective measures or mixed-methods approaches. While efforts were made to mitigate common method variance (CMV), residual CMV may persist. To address this, future research could employ diverse data sources to minimize bias. Lastly, this study focused on individual-level analysis, overlooking potential hospital system influences. Future investigations could incorporate hospital-level factors for a more comprehensive understanding. 

## 7. Conclusions

Our study emphasizes the crucial role of participation motivation within medical teams in facilitating interdisciplinary collaborative care. We found that medical teams with higher levels of participation motivation are more open to the suggestions and expertise of medical staff from various fields. This receptiveness enables them to successfully integrate interdisciplinary approaches into medical care, ultimately leading to enhanced patient outcomes.

Moreover, our research underscores the importance of investment in medical services by motivated medical teams. Their willingness to dedicate time, effort, and resources to patient care significantly contributes to the delivery of high-quality healthcare services. By embracing interdisciplinary collaboration and prioritizing patient-centered care, these motivated medical teams ensure that patients receive comprehensive and holistic care tailored to their individual needs.

Looking ahead, it is essential for healthcare organizations to acknowledge and foster participation motivation among medical teams. Cultivating a culture of collaboration, continuous learning, and mutual respect within healthcare institutions can enhance the effectiveness of interdisciplinary care delivery models. Additionally, future research should explore strategies to sustain and reinforce participation motivation among medical professionals, thereby further optimizing patient care and outcomes.

## Figures and Tables

**Table 1:** Description of respondents.

Measure		Frequency *N* = 324	Percentage (%)
Hospitalist system model			
	Total care physicians	73	22.5
	Mixed care model for inpatient care	251	77.5
Hospital accreditation level			
	Medical center	231	71.3
	Regional hospitals	93	28.7
Age	Mean age was 38.1 years (standard deviation: 9.3 years)		
Gender	Women	310	96.7
	Male	14	4.3
Marital status	Single	194	59.9
	Married	122	37.7
	Widowed (divorced)	8	2.5
Level of education	College	34	10.5
	Bachelor’s degree	273	84.3
	Postgraduate (masters/doctoral)	17	5.2
Supervisor	No	256	79
	Yes	68	21
Health professional			
	Attending physician	42	13.0
	Registered nurse	114	35.2
	Other healthcare professional	167	51.8

**Table 2:** Measurements and confirmatory factor analysis.

Construct	Number of Questions	Mean	Standard Error	Cronbach’s Alpha	Average Variance Extracted	Construct Reliability
Awareness	13	4.365	0.560	0.982	0.5884	0.9489
Participation motivation	8	4.399	0.563	0.979	0.4145	0.8496
Participation behavior	8	4.385	0.569	0.973	0.3209	0.7902
Value creation	15	4.324	0.553	0.980	0.5282	0.9433

**Table 3:** Overall fit of the research model.

Model-Fit Index	Recommended Value	Score
Chi-square/degree of freedom (χ^2^/df)	≤3	2.394
Goodness-of-fit index (GFI)	≥0.8	0.882
Comparative fit index (CFI)	≥0.9	0.942
Normed fit index (NFI)	≥0.9	0.941
Tucker–Lewis Index (TLI)	≥0.9	0.901
Root Mean Square Error of Approximation (RMSEA)	≤0.08	0.036
Incremental fit index (IFI)	≥0.9	0.942

**Table 4:** Structural model results.

Hypothesis	Path	Path Coefficient	t-Value	*p*-Value	Findings
H1	Awareness → Participation motivation	0.846	28.427	<0.001	Supported
H2	Participation motivation → Participation behavior	0.888	34.620	<0.001	Supported
H3	Participation behavior → Value creation	0.869	31.512	<0.001	Supported
H4	Awareness → Value creation	0.782	22.494	<0.001	Supported

## Data Availability

Data cannot be made publicly available owing to the fact that the privacy of individual participants cannot be compromised. However, the dataset is available from the corresponding author on reasonable request.

## Appendix A. Questionnaires

Holistic Health Care Awareness Questionnaire (13 questions)

1. Physical comfort provision in holistic health care.
2. Comprehensive consideration of patients’ needs in holistic health care.
3. Emotional support provision in holistic health care.
4. Addressing patients’ anxiety, fear, and privacy in holistic health care.
5. Involving patients and families in understanding and decision-making.
6. Understanding socio-economic needs in holistic health care.
7. Providing accessible, continuous, coordinated, and comprehensive treatment plans.
8. Providing spiritual care in holistic health care.
9. Offering preventive, health promotion, acute, chronic, long-term, rehabilitation, and end-of-life care.
10. Considering patients’ family, lifestyle, and economic status.
11. Respecting opinions of patients, caregivers, and family in treatment.
12. Educating healthcare professionals adequately in holistic care.
13. Interprofessional collaborative practice, division of labor, and communication.

Hospitalist System Participation Motivation Questionnaire (8 questions)

1. Establishing common goals in interprofessional collaborative practice.
2. Cooperation and resource sharing in interprofessional collaborative practice.
3. Coordination of medical decisions and activities in interprofessional collaborative practice.
4. Increasing professional knowledge through interprofessional collaborative practice.
5. Increasing learning opportunities in interprofessional collaborative practice.
6. Providing interaction and communication opportunities with others in interprofessional collaborative practice.
7. Collaboratively solving patient care problems in interprofessional collaborative practice.
8. Addressing medical and social resource needs of patients in interprofessional collaborative practice.

Hospitalist System Participation Behavior Questionnaire (8 questions)

1. Resolving multiple patient diseases through interprofessional collaborative practice.
2. Reducing waste of medical resources through interprofessional collaborative practice.
3. Improving medical quality through interprofessional collaborative practice.
4. Achieving team identity through interprofessional collaborative practice.
5. Sharing responsibility through interprofessional collaborative practice.
6. Assessing patient problems at various levels through interprofessional collaborative practice.
7. Improving medical cooperation quality for patients, families, and communities through interprofessional collaborative practice.
8. Providing cooperation participation and expertise value creation through interprofessional collaborative practice.

Value Creation Questionnaire (14 questions)

1. Providing comprehensive medical care through interprofessional collaborative practice.
2. Feeling respected through interprofessional collaborative practice.
3. Reducing anxiety in patient care and enabling positive thinking through interprofessional collaborative practice.
4. Improving relationships with colleagues through interprofessional collaborative practice.
5. Reducing medical disputes for patients through interprofessional collaborative practice.
6. Increasing job satisfaction through interprofessional collaborative practice.
7. Fostering team leadership skills through interprofessional collaborative practice.
8. Enhancing doctor-patient relationship harmony through interprofessional collaborative practice.
9. Improving patient satisfaction through interprofessional collaborative practice.
10. Enhancing overall patient safety through interprofessional collaborative practice.
11. Improving overall healthcare quality through interprofessional collaborative practice.
12. Reducing hospital medical dispute cases through interprofessional collaborative practice.
13. Creating lean healthcare for hospitals through interprofessional collaborative practice.
14. Enhancing overall employee satisfaction through interprofessional collaborative practice.
